# Host Response Comparison of H1N1- and H5N1-Infected Mice Identifies Two Potential Death Mechanisms

**DOI:** 10.3390/ijms18081631

**Published:** 2017-07-27

**Authors:** Olivier Leymarie, Léa Meyer, Pierre-Louis Hervé, Bruno Da Costa, Bernard Delmas, Christophe Chevalier, Ronan Le Goffic

**Affiliations:** 1 VIM, INRA, Université Paris-Saclay, Jouy-en-Josas, Paris 78350, France; olivier.leymarie@gmail.com (O.L.); lea.meyer@inra.fr (L.M.); pierre-louis.herve@dbvtechnologies.onmicrosoft.com (P.-L.H.); bruno.da-costa@inra.fr (B.D.C.); bernard.delmas@inra.fr (B.D.); christophe.chevalier@inra.fr (C.C.); 2 Département de Virologie, Unité de Génétique Moléculaire des Virus à ARN, Institut Pasteur, Paris 75015, France

**Keywords:** influenza virus, host response, mouse model, transcriptome, H5N1, H1N1

## Abstract

Highly pathogenic influenza A viruses (IAV) infections represent a serious threat to humans due to their considerable morbidity and mortality capacities. A good understanding of the molecular mechanisms responsible for the acute lung injury observed during this kind of infection is essential to design adapted therapies. In the current study, using an unbiased transcriptomic approach, we compared the host-responses of mice infected with two different subtypes of IAV: H1N1 vs. H5N1. The host-response comparison demonstrated a clear difference between the transcriptomic profiles of H1N1- and H5N1-infected mice despite identical survival kinetics and similar viral replications. The ontological analysis of the two transcriptomes showed two probable causes of death: induction of an immunopathological state of the lung for the H1N1 strain vs. development of respiratory dysfunction in the case of the H5N1 IAV. Finally, a clear signature responsible for lung edema was specifically associated with the H5N1 infection. We propose a potential mechanism of edema development based on predictive bioinformatics tools.

## 1. Introduction

Influenza A virus (IAV), the causative agent of flu, is a major pathogen responsible for epidemics and pandemics worldwide. IAV is an enveloped virus belonging to the Orthomyxoviridae family, its genome consists of 8 negative-sense RNA segments encoding up to 17 proteins [1]. Every year, IAV causes between 250,000 and 500,000 deaths around the world [2] and is therefore considered a major threat to public health.

In humans, IAV mainly infects airway epithelial cells. The infection induces an inflammatory response leading to rapid recruitment of monocytes and neutrophils to the lungs [3]. IAV can cause mild to severe respiratory illness, and can result in death. Acute pneumonia is a severe complication of IAV mainly attributable to highly pathogenic (HP) strains, such as H5N1. HP strains trigger severe damage to the lungs, and consequently tremendous amounts of proinflammatory cytokines and chemokines are produced by IAV infected cells. This uncontrolled inflammatory cascade is called a “cytokine storm” or “hypercytokinemia” and often leads to death [4].

The pathogenesis mediated by HP strains is therefore very different from the one observed during infection by seasonal IAV (i.e., human H1N1 and H3N2 strains). Those differences are visible through histology of infected-mice, the HP IAV infection harboring very characteristic histological features [5]. In addition to this specific histological signature, HP IAV causes a different course of acute respiratory distress syndrome establishment in comparison to seasonal viruses.

Mouse remains a useful mammalian model of IAV infection despite several limitations [6]. Indeed, the mouse model is not adapted to study viral transmission [7] as infected-mice do not shed virus from the respiratory tract. However, in terms of immune response, mice share similarities with cynomolgus macaques [8] and are currently used to provide important insights into the pathogenesis mechanisms induced by IAV and HP IAV [9]. In addition, the mouse model is very useful to decipher the differences of pathological mechanisms induced by different virus strains as illustrated in the comparative study by Otte and collaborators [10], in which they identified host determinants responsible for pathogenesis in different type of mice.

Avian strains of IAV are capable of crossing the species barrier to infect mammals. In recent decades, several zoonotic avian infections of humans have been documented [11]. The incriminated viruses usually belong to the H5N1 strain, but also to H7N7, H7N3, H9N2, and more recently H7N9 strains [12]. These avian IAVs display different features compared to mammalian strains, including transmissibility and pathogenicity differences. All these differences can be explained at the molecular level and can be characterized by studying the host response.

In the present study, we used the mouse model to compare the host response during H1N1 and H5N1 lethal infections. The H1N1 (WSN) virus is a mouse-adapted lab strain inducing an acute pathology in the mouse model and the H5N1 IAV is an avian HP strain. We hypothesized that, due to their different origins and despite similar survival curves and comparable virus replication kinetics, H1N1 and H5N1 viruses induce different pathological profiles in mice. We used an unbiased transcriptomic approach to characterize the infection profiles in mice and found distinct H1N1- and H5N1-induced mechanisms leading to death.

## 2. Results

### 2.1. Establishment of Lethal Model of H1N1 and H5N1 Infections in Mice

**Figure 1:**
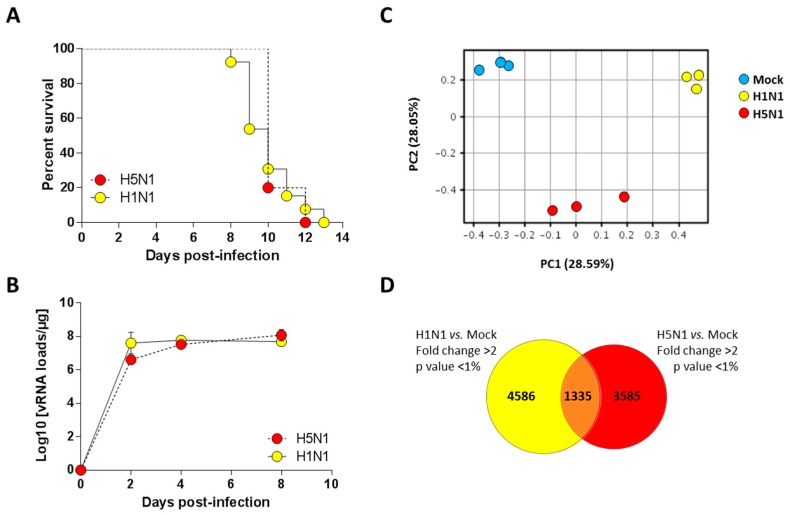
Survival curves and viral loads of infected mice. (**A**) Lethalities of H5N1 and H1N1 were compared in mice infected with 1.4 × 10^2^ PFU and 1.5 × 10^5^ PFU of H5N1 (*n* = 10) or H1N1 (*n* = 13), respectively; (**B**) Viral load in the lungs of H5- and H1-infected mice were measured using quantitative RT-PCR and expressed as copy numbers per µg of total lung RNA. Results are the mean ± SD obtained from six mice; (**C**) Principal component analysis of the lung genes normalized expression values. X-axis represents the first dimension (Component 1, 28.59% of the variance) which separates the mock conditions from the infected conditions, the y-axis represents the second dimension (Component 2, 28.05% of the variance) which separates the viral infections (i.e., H1N1 vs. H5N1); (**D**) Venn diagram depicting the differentially expressed genes (≥2.0-fold change, *p*-value < 0.05) common or unique to H1N1- and H5N1-infected mice relative to mock-infected mice.

We previously characterized a H1N1 (A/WSN/1933) and a H5N1 (A/duck/Niger/2090/2006) infection model in the C57Bl/6 mouse [13,14]. We took advantage of these two models to compare the host response of the mice to both influenza viruses. We used a dose of 1.5 × 10^5^ PFU of H1N1 virus and a dose of 1.4 × 10^2^ PFU of the H5N1 virus to perform a lethal challenge (corresponding to around 10 MLD50 (mouse lethal dose 50)). As shown in Figure 1A, the two groups of infected mice died at comparable kinetics with a median survival of 10 days post-infection (pi). Interestingly, the viral loads measured within the airways demonstrated that kinetics of viral growth were very similar in both types of infection despite different inoculum. Both viruses showed robust replication patterns, with viral RNA levels increasing consistently up to four days pi before reaching a stationary phase. Viral RNA levels were nearly equivalent for both viruses at day 4 pi (Figure 1B). Thus, we chose this time point to analyze and compare global gene expression patterns in mice lungs in response to infection.

### 2.2. Transcriptomic Profiling of Infected Lungs

In order to finely analyze and characterize the responses to infection by H1N1 and H5N1 IAV, we compared the mRNA expression profiles of H1N1- or H5N1-infected mouse lung tissues at four days pi to mRNA from mock-infected animals. Importantly, the cellular tropisms of the two viruses were examined using immunohistochemistry and showed a similar robust replication within the airway epithelium (data not shown). Principal component analysis was performed to represent the variance in the gene dataset. The score plot presented in Figure 1C revealed three clusters totally independent using the first two components (PC1 and PC2) that represent 28.59% and 28.05% of the variance, respectively. The three gene clusters are totally separated following PC1 and PC2 axis, indicating different gene expression profiles between mock and infected conditions, but also between the two different viral infections. Among the 44 × 10^3^ probes present on the microarrays, 5921 and 4920 probes were differentially regulated in H1N1- and H5N1-infected mouse lung tissues, respectively (Figure 1D). Surprisingly, only 1335 probes were commonly regulated by the two viruses. Collectively, our data suggest that despite similar mortality kinetics and replication rates, the H1N1 and H5N1 IAV induced a different host response that could reflect different mechanisms leading to death.

### 2.3. Expression Clustering and Gene Ontology

**Figure 2:**
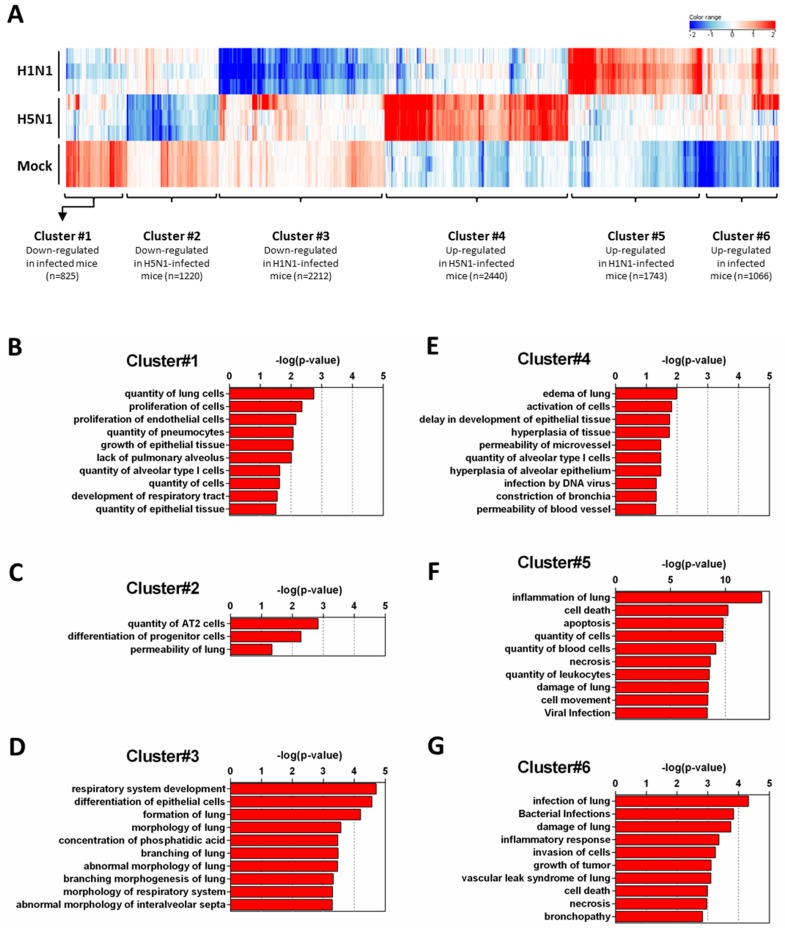
Functional relationships of pathways regulated during infection. Total RNA from mock-, H1N1-, or H5N1-infected C57Bl/6 mice were extracted from lungs at day 4 pi and used to hybridize Agilent’s Whole Mouse Genome Microarray (4 × 44K; G4122F). GeneSpring software (Version 13.0; Agilent Technologies) was used to analyze differences of gene expression between mock-infected mice and H1N1- or H5N1-infected mice. (**A**) Hierarchical clustering diagrams showing individual replicates are represented. The magnitude of the expression is illustrated by the intensity of the color. Red and blue indicate higher and lower expression level, respectively; (**B**–**G**) Molecular and cellular functions associated with differentially expressed genes were compared using IPA. Each cluster was separately analyzed and annotations were ranked using the *p*-value obtained using the right-tailed Fisher’s exact test, it represents the probability of each biological function to be involved in the group of analyzed genes.

In order to get a comprehensive view of the host responses, we conducted a density cluster analysis on all the differently expressed genes at four days pi to determine groups of genes with similar expression profiles. Hierarchical clustering analysis was performed to identify cellular genes that were differentially expressed during H1N1 and H5N1 infections (similarity measure: Euclidean; linkage rules: Wards). We obtained six distinct clusters: three clusters exhibiting downregulated genes and three clusters exhibiting upregulated genes compared to the mock-infected condition (Figure 2A). To explore the functional consequences of gene expression changes associated with virus infection, we uploaded the gene ID composing the six differentially expressed clusters in the IPA software in order to identify and classify the functions associated to these groups of genes.

**Figure 3:**
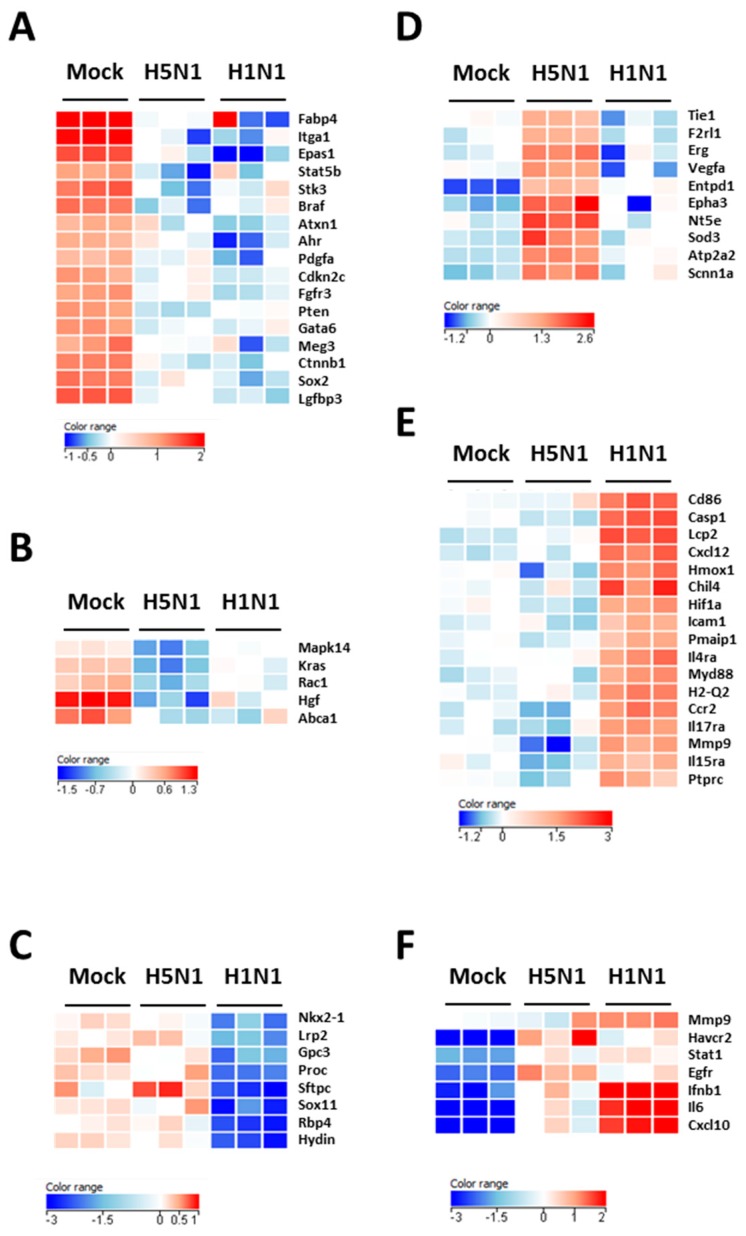
Heat maps of selected genes in the lungs of mock- H1N1- and H5N1-infected mice at day 4 pi. Representative genes of each cluster were selected and represented as a heat map. Genes shown in red are upregulated and genes in blue are downregulated. The magnitude of the regulation is illustrated by the intensity of the color. (**A**) Heat map of genes from cluster 1 and related to growth of epithelial tissue; (**B**) Heat map of genes from cluster 2 and related to quantity of AT2 cells; (**C**) Heat map of genes from cluster 3 and related to respiratory system development; (**D**) Heat map of genes from cluster 4 and related to edema of lung; (**E**) Heat map of genes from cluster 5 and related to inflammation of lung; (**F**) Heat map of genes from cluster 6 and related to infection of lung.

Figure 2B–D show the downregulated clusters 1, 2, and 3, respectively. Strikingly, in spite of a difference in gene composition, the three downregulated clusters displayed a transcriptional enrichment in very similar functions during infection by the two viruses: growth, development, differentiation and morphology of the respiratory epithelium (Figure 2B–D and Figure 3A–C). Interestingly, cluster 1 which represents the genes downregulated by both viruses (Figure 2B) was particularly enriched in gene related to growth of epithelial and endothelial cells. These gene signatures reflected a strong impact of virus infections on epithelial structure and suggested a stop in epithelial cell turnover, illustrating a deep morphological changes induced during the viral infections. 

**Figure 4:**
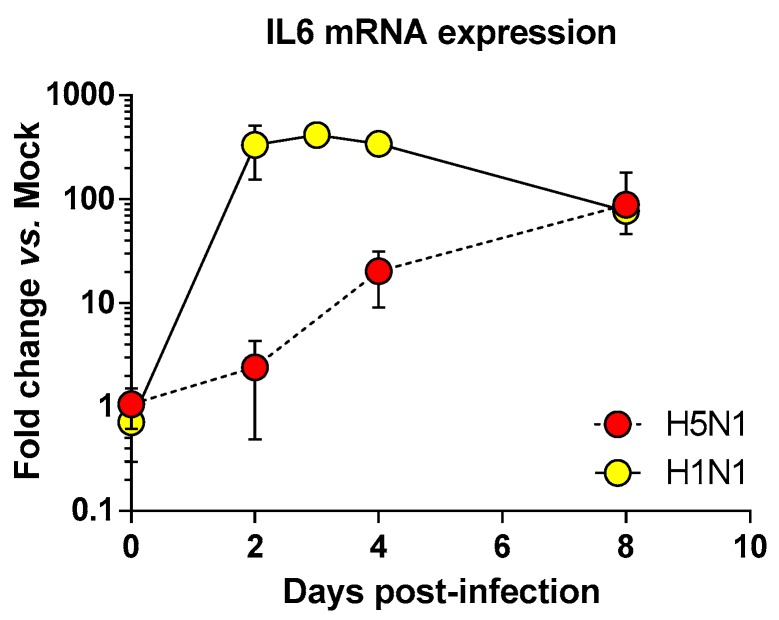
Time course of IL6 mRNA expression in the airways of infected mice. Mice were infected with 1.4 × 10^2^ PFU and 1.5 × 10^5^ PFU of H5N1 or H1N1 respectively and were euthanized at two, four, and eight days pi. After extraction, lung total RNAs were reverse transcribed and used to quantify IL6 transcription. IL6 mRNA levels were normalized to β-actin mRNA levels and presented as fold increase relative to mock-treated mice.

We next focused on the genes upregulated by the IAV infections: cluster 4 (upregulated in H5N1-infected mice; Figure 2E), cluster 5 (upregulated in H1N1-infected mice; Figure 2F), and cluster 6 (upregulated in both IAV-infected mice; Figure 2G). When examining the cellular genes commonly induced during both viral infection (i.e., cluster 6), the ontological analysis showed a clear host defense signature: infection of lung and inflammatory response associated with a pathological signature: damage of lung, necrosis, bronchopathy (Figure 2G). This cluster comprised 1066 genes including Ifnb1, Stat1, Il6, and Cxcl10 (Figure 3F). The same type of response is observed for cluster 5 (upregulated in H1N1-infected mice): inflammation of lung, cell death, necrosis, and damage of lung. This suggests that, at this time point, the signature related to host response and pathogenesis plays a more important role in H1N1-infected mice in comparison to H5N1-infected mice. The pronounced inflammatory response characterizing the H1N1 infection is illustrated in the heat map presented in Figure 3E. In order to validate the microarray expression data, we quantified the IL6 mRNA expression during the course of infection by the 2 IAV. As shown in Figure 4, the kinetic of expression of this major inflammatory cytokine clearly shows differences of expression depending on the type of the virus. The level of expression of IL6 is 10 to 100 times more important during the early steps of H1N1 infection (i.e., between two and four days pi), confirming the major inflammation of the H1N1-infected lungs.

To explore the H5N1-associated host responses, we then focused our analysis on the cluster 4 which represents a group of genes specifically induced during H5N1 infection (Figure 2E). Notably, cluster 4 was enriched in several genes linked to a typical lung edema signature including Vegfa, Tie1, Sod3, Nt5e, Scnn1a (Enac), F2rl1 (Par2), Erg, Epha3, Entpd1, and Atp2a2 (Serca2) (Figure 3D). Pulmonary edema is defined as an accumulation of extravascular fluid in the alveolar compartment and its clearance is mainly driven by the Na^+^/K^+^ transporting ATPases, which promote vectorial ions transfer and subsequently alveolar fluid resorption following the osmotic gradient [15]. Cluster 4 also contained genes associated with permeability of blood vessel or microvessel: Creb1, Mif, Pik3r1, and Tacr1 (data not shown), a process linked to edema. Of note, the molecular and cellular functions related to cluster 4 also include constriction of bronchi, a reflex constriction of airway smooth muscle frequently observed during inflammation of the lungs [16]. Importantly, all these genes are not regulated by the H1N1 virus and therefore represent a specific signature of the H5N1 infection.

Collectively, our transcriptomic comparison of H1N1- and H5N1-infected mice clearly shows two different types of response. The H1N1-specific potent inflammatory response resembles a phenomenon called hypercytokinemia, also known as “cytokine storm”, whereas the H5N1-specific response relates to a pathological respiratory response typically observed during lung edema.

### 2.4. Prediction Model

**Figure 5:**
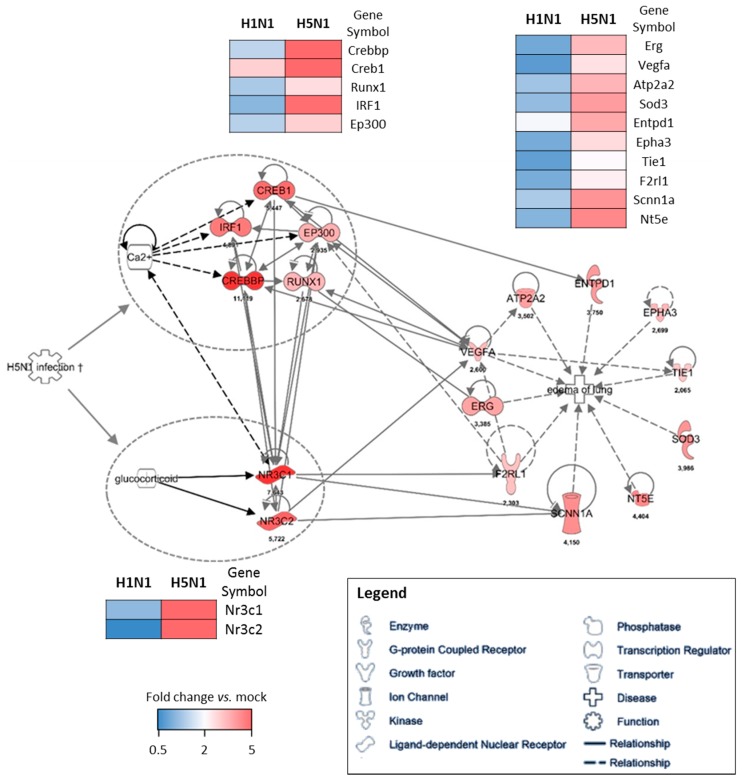
Gene–gene interaction networks of the edema signature. IPA was used to enrich the gene network with possible upstream regulators. Direct interaction between two genes is shown as a solid line and indirect interaction is depicted as a dashed line (based on literature reports). The fold change expression of H5N1-infected mice vs. mock-infected mice is indicated at the bottom of each node. The comparison of the mean level of expression (fold change vs. mock) of each gene between H1N1 and H5N1 infected condition is represented as heat maps.

To further investigate the potential regulation mechanisms of the genes responsible for the edema signature, we used the IPA software as a predictive tool to identify the upstream regulatory element. We used the cluster of genes upregulated in H5N1-infected mice (cluster 4) as a data set to perform an “upstream analysis” by focusing on transcription factors. We identified two groups of genes with transcriptional activities that were highly connected to the genes composing the edema signature, which means they might contribute to its regulation (Figure 5). Two nuclear receptors activated by glucocorticoids, NR3C1 and NR3C2 (nuclear receptor subfamily 3 group C member 1 and 2), represent one of the two potential regulatory elements of the edema signature. They are highly associated to Vegfa, F2rl1 (Par2), and Scnn1a (Enac) and their expression levels highly discriminate H5N1 from H1N1 infection. Of note, the growth factor Vegfa appears to be a hub in this gene network and therefore likely orchestrates the development of edema during the H5N1 infection. The second regulatory element is made of five interconnected transcription factors: Irf1, Creb1, Crebp, Ep300, and Runx1. Irf1 is an interferon regulatory factor and is logically induced during the infection, however its expression is not induced during the H1N1 infection. The other four transcription factors are also very specific to H5N1 infection and are tightly linked to the edema signature. Their activity appears to be tightly dependent on the Ca^2+^ concentration, which could therefore be a link between H5N1 infection and the induction of this group of transcription factors.

## 3. Discussion

In the present study, we performed a comparison of the host responses developed by mice infected by two different HP IAV: a mouse-adapted H1N1 strain and an avian H5N1 strain. We report two differential pathogenic mechanisms used by two HP IAVs, and leading to death. The comparison was possible due to similar mortality and morbidity kinetics and because the replication activities of the two viruses were very comparable. The viral loads were identical at day 4 pi, the time point chosen to perform the analysis. Despite similar symptoms leading to the death of infected-animals, host responses against the two pathogens exhibited important differences in terms of both intensity and functionality. Our data demonstrate that the immunopathology developed in response to the viral aggressor is based on an inflammatory response that leads to two different pathologic pathways: a cytokine storm in the case of the H1N1 virus regrouping signatures for leukocyte recruitment, cell death, or necrosis (Figure 2F); and a respiratory dysfunction in the case of H5N1 virus, including expression of genes implicated in hyperplasia of alveolar epithelium or in constriction of bronchia (Figure 2E).

During IAV infection, the pro-inflammatory response is critical in order to mobilize the immune actors to the site of infection. In the case of HP IAV infections, this response is frequently exacerbated and gives rise to a detrimental feedforward inflammatory response responsible for death [17]. This typical damaging innate immune response is observed in our H1N1 specific signature (cluster 5, Figure 2F) and is probably at the origin of the death of the mice as previously described [9,18]. It must be noted that the implication of the hypercytokinemia in the cause of the death is a matter of debate [19,20]. In the case of the H5N1 infection, the early immune response appeared very inhibited in comparison to the H1N1 infection. It is probable that the NS1 protein from H5N1 could be very efficient in the suppression of host immune response.

Our comparison analysis showed a clear lung edema signature discriminating the H5N1 infection from the H1N1 infection. Pulmonary edema is characterized by fluid accumulation within the lungs contributing to airways obstruction and hypoxia. In humans, edema is a hallmark of acute respiratory distress syndrome (ARDS), and is frequently observed in severe case of IAV infection. The development of pulmonary edema during HP IAV (H5N1) infection has been considered a poor prognostic factor for patients developing an IAV-induced ARDS [15]. Clearance of edema fluid is therefore critical for the survival of the patient with acute lung injury.

Given the edema signature that appears very specific for our H5N1 strain, we used a combination of bioinformatic predictive tools to identify the regulatory elements that could lead to such a signature. This methods allowed us to identify two potential groups of transcription factors highly connected to the genes composing the edema signature. The first group of genes is NR3C1 and NR3C2, which encode nuclear receptors with transcription factor activities. They are activated upon binding to glucocorticoids and have been described to exert a role in ion and water transport and thus raise extracellular fluid volume and pressure [21]. In addition, NR3C2 has also been shown to act on the Renin–Angiotensin–Aldosterone pathway, and is therefore in close interaction with Na+, K+, and H+ homeostasis [22]. Given their implication in water retention and electrolyte transport and the fact that they are specifically induced by H5N1, these two NR3C receptors are consequently good candidates to explain the H5N1-related edema of lung signature identified in this study.

The second group of transcription factors predicted to be implicated upstream of the edema signature are composed of three Creb members (Creb1, Crebbp, and Ep300), and of Runx1 (runt related transcription factor 1) and Irf1. This group of transcription factors forms a small interconnected network regulated by second messenger such as cAMP or Ca^2+^. Creb expression is essential in lung physiology and mice lacking Creb have been described to die postnatally due to impairment of lung function [23]. Interestingly, Creb is also implicated in increased endothelial permeability during acute inflammatory lung injury [24], a process that implicates protease activated receptors (Par, here named F2rl1) and Vegf. Creb is indeed required for restoring lung vascular barrier function. Given the interaction of Irf1 and Creb transcription factors, it is thus possible to speculate that the antiviral signaling (or another IAV-induced inflammatory signal) could prevent the resolution of the alveoli-capillary damage mediated by the Creb transcription factors. Ultimately, this would result in fluid leakage and persistent edema formation leading to severe oxygenation impairment and death of the mice.

In conclusion, the transcriptomic approach to study the host-response against IAV constitutes a powerful method not only to characterize pathogen virulence, but also for studying pathological mechanisms. Our future challenge will be to decipher which viral determinants are associated to a certain kind of pathology and to define specific markers to adapt therapeutics strategies. 

## 4. Materials and Methods

### 4.1. Mice

Female C57BL/6J mice were purchased from the Centre d’Elevage R. Janvier (Le Genest Saint-Isle, France) and were used at about eight weeks of age. Mice were lightly anesthetized with a mixture of ketamine and xylazine (60 mg/kg and 12 mg/kg respectively) and were inoculated intranasally with 1.5 × 10^5^ or 1.4 × 10^2^ PFU of H1N1 or H5N1 virus respectively in 50 µL PBS. Mice were observed daily for signs of morbidity and euthanized at different time points by cervical dislocation. After euthanasia, lungs were aseptically removed, rinsed twice with PBS, and frozen in dry ice for further RNA extraction. This study was carried out in accordance with INRA guidelines in compliance with European animal welfare regulation. The protocols were approved by the Animal Care and Use Committee at “Centre de Recherche de Jouy-en-Josas” (COMETHEA) under relevant institutional authorization (“Ministère de l’éducation nationale, de l’enseignement supérieur et de la recherche”), authorization number: 2015100910396112v1 (APAFIS#1487).

### 4.2. Viruses

Influenza viruses A/WSN/1933 (H1N1) and A/duck/Niger/2090/2006 (H5N1) were produced using reverse genetic as previously described [13,14,25,26]. The viruses were titrated on Madin Darby Canine Kidney (MDCK) cells.

### 4.3. RNA Extraction and Microarray Experiments

Total RNA was extracted from lung samples using RNeasy kit (Qiagen, Courtaboeuf, France) and were treated with DNase I. Lung transcriptional profiling was performed using Agilent’s Whole Mouse Genome Microarray Kit, 4 × 44K (G4122F). Minimum Information about Microarray Experiment (MIAME) was deposited in ArrayExpress at EMBL (http://www.ebi.ac.uk/microarray-as/ae). Arrays were hybridized according to the manufacturer’s instructions and as previously described [13,14,27]. For functional analysis the data files resulting from differential analysis were imported into GeneSpring software (Version 13.0; Agilent Technologies, Massy, France). Hierarchical clustering analysis was performed to analyze cellular genes that were differentially expressed during infection (Canberra distance, centroid linkage). For further analysis, data files were uploaded into the Ingenuity Pathways Analysis (IPA) software (Ingenuity Systems, Redwood, CA, USA; www.ingenuity.com).

### 4.4. Statistical Analysis

Principal component analysis was performed on normalized data using the GeneSpring software (Version 13.0; Agilent Technologies). The data were log transformed and median normalized. Expression values obtained were submitted to a Student’s unpaired *t*-test, and *p* values were adjusted using the Benjamini–Hochberg multiple testing correction. Significant probes with a Benjamini–Hochberg *p* < 0.01 and fold change ≥2 were used for ontological analysis. Ontological analysis was made with IPA and used the right-tailed Fisher’s exact test to calculate a *p*-value determining the probability that each biological function and disease assigned to that data set is due to chance alone.

## Abbreviations

IAV	Influenza A virus
HP	Highly pathogenic
Pi	Post-infection
ARDS	Acute respiratory distress syndrome
MLD50	Mouse lethal dose 50
